# Person-centered maternity care and its associated factors during childbirth at selected public hospitals in Eastern Ethiopia: a cross-sectional study

**DOI:** 10.3389/fgwh.2025.1513808

**Published:** 2025-09-11

**Authors:** Arsema Gebreyesus, Agumasie Semahegn, Fikru Tebeje, Arega Abebe Lonsako, Sagni Girma

**Affiliations:** 1 School of Nursing and Midwifery, College of Health and Medical Sciences, Haramaya University, Harar, Ethiopia; 2 Center for Innovative Drug Development and Therapeutic Trials for Africa (CDT-Africa), College of Health Science, Addis Ababa University, Addis Ababa, Ethiopia; 3 School of Medical Laboratory Science, College of Health and Medical Sciences, Haramaya University, Harar, Ethiopia; 4 School of Nursing, College of Medicine and Health Science, Arba Minch University, Arba Minch, Ethiopia; 5 Department of Obstetrics and Gynecology, Leiden University Medical Centre, Leiden, Netherlands

**Keywords:** person-centered maternity care, women, childbirth, public hospitals, Ethiopia

## Abstract

**Background:**

Maternal mortality has remained a major public health issue globally. Although there has been substantial reduction in maternal mortality, Ethiopia is still one of the highest burden countries in sub-Saharan Africa. Person-centered maternity care plays a key role in ending preventable maternal mortality. Nevertheless, little is known about the status of person-centered maternity care during facility-based childbirth in eastern Ethiopia. Therefore, the aim of this study was to assess the status of person-centered maternity care and its associated factors during childbirth at selected public hospitals in eastern Ethiopia.

**Methods:**

We had conducted a facility-based cross-sectional study at selected public hospitals in eastern Ethiopia from May 16 to June 17, 2022. A total of 420 postpartum women, selected by a systematic random sampling technique, were included in the study. We had collected our data by face-to-face interview using a pretested structured questionnaire. Then, the data were entered into EpiData 4.6 and exported to SPSS version 26 for cleaning and analysis. We applied linear regression analyses to determine the associations between dependent and independent variables. The association was reported using a β coefficient with a 95% confidence interval (CI) and a *p*-value ≤0.05.

**Results:**

The percentage mean score of person-centered maternity care was 68.1 (CI: 59.94, 62.66), SD (±14.1). From the subscales of person-centered maternity care, the percentage mean score of dignity and respect was 80.6%, communication and autonomy 61.1%, and 67.3% for supportive care. Women who'd had antenatal care (ANC) follow-up (*β* = 5.66, 95% CI: 2.79, 8.53) and women who gave birth to a live newborn (*β* = 7.59, 95% CI: 3.97, 11.20) had a positive association with person-centered maternity care. However, women who had experienced childbirth complications (*β* = −7.01, 95% CI: −9.88, −4.13) and those who had a hospital stay of more than two days (*β* = −4.08, 95% CI: −6.79, −1.38) were negatively associated with person-centered maternity care.

**Conclusion:**

Our study revealed that the mean person-centered maternity care score of the participants was significantly higher than in previous studies. Women who had antenatal care follow-up, experienced complications during childbirth, gave birth to a live newborn, and had a hospital stay of more than two days were significantly associated with person-centered maternity care. Therefore, we strongly concluded that strengthening antenatal care utilization and early detection and appropriate management of childbirth and pregnancy complications would greatly improve person-centered maternity care.

## Introduction

Despite a 38% reduction in the maternal mortality ratio (MMR) between 2000 and 2017, maternal deaths remained a global public health challenge (1). Every day in 2020, about 800 women had died from preventable causes, with 94% of these deaths having occurred in low and middle-income countries (2). In Ethiopia, although maternal mortality has been reduced by half since 2000, it is still estimated to be 412 deaths per 100,000 live births (3). Maternal deaths in Ethiopia have been attributed to preventable causes such as postpartum complications, home delivery, and abortion, among others (1). Poor quality of care is a major factor in maternal deaths and is a significant barrier for women who seek healthcare services. Person-centered maternity care is one aspect of quality care that needs to be addressed.

Person-centered care is when a person can be the driving force of their own healthcare decisions and receive healthcare that is tailored to their own values and preferences. It is a fundamental concept that guides the setting of care philosophy from a traditional biomedical model to a more humanistic approach (2). It is considered a gold standard dimension of quality care and a major theme in modern healthcare systems (3).

Person-centered maternity care (PCMC) is providing respectful and responsive care that is specifically tailored to an individual woman's preferences, values, and needs during childbirth (4). The World Health Organization has fully recognized PCMC as a key component of quality maternity care (5). Dignity and respect, communication and autonomy, and supportive care are identified as fundamental components of PCMC (5).

PCMC prioritizes the quality of a woman's birth experience by encouraging her to feel free, safe, and confident enough to express her feelings and needs to the healthcare provider (6). It is a strong health promotion approach that increases women's satisfaction, decreases anxiety, and improves healthcare utilization (7). According to existing evidence, there is a significant association between PCMC and women's positive childbirth experiences (8). PCMC has been associated with lower neonatal complications and a higher willingness of women to return to the health institution for their future childbirth (9, 10). Due to the evidence presented above, it is quite clear that PCMC is crucial for the improvement of both maternal and neonatal health. As a result, global movements have called for greater emphasis on PCMC (11).

Every woman deserves self-centered, dignified, and respectful maternal care during childbirth (12). However, thousands of women are facing various forms of mistreatments during childbirth in many parts of the world (13). According to a study conducted in East and Southern Africa, many women had poor interactions with providers, noting that the procedures or care they received were not adequately explained to them. Additionally, both physical and verbal abuses were also reported by the participants (14).

Poor quality care during childbirth significantly contributes to maternal deaths. The lack of PCMC has been associated with maternal complications such as obstructed labor and postpartum hemorrhage (15). When maternity care is not centered on the needs and preferences of the woman and she feels unheard, she may be less likely to report concerning symptoms or ask questions about her care. This lack of open dialogue can hinder the provider's ability to monitor and respond promptly to emerging complications including, obstructed labor, postpartum hemorrhage, birth canal lacerations, increased risk of cesarean section, and fetal distress (15–17). Therefore, PCMC plays a critical role in decreasing both maternal morbidity and mortality (6). It is also a significant strategy for achieving the Sustainable Development Goal of reducing maternal mortality to less than 70 deaths per 100,000 live births (18).

Furthermore, studies conducted in Ethiopia had revealed that two-thirds of women reported their healthcare providers had never introduced themselves and that they gave birth without a birth companion (19). Additionally, 34.5% of women had often been slapped by their healthcare provider during delivery (20). Nevertheless, there is limited research evidence regarding the status of PCMC in eastern Ethiopia, so the aim of this study was to assess the status of person-centered maternity care and its associated factors during childbirth at selected public hospitals in eastern Ethiopia.

## Materials and methods

### Study setting and design

We conducted a facility-based cross-sectional study from May 16 to June 17, 2022, at purposively selected three public hospitals in eastern Ethiopia, namely: Hiwot Fana Comprehensive Specialized University Hospital (HFCSUH), Haramaya General Hospital (HGH), and Dil Chora Referral Hospital (DCRH). HFSCUH is a comprehensive teaching hospital affiliated with Haramaya University College of Health and Medical Sciences, which is located in Harar town at a distance of 526 km to the east of Addis Ababa, the capital city of Ethiopia. It serves around 5.8 million people of the surrounding population (21). The Haramaya General Hospital is located in Haramaya town, at a distance of 507 km to the east of Addis Ababa. It serves about 1,143,909 Haramaya district residents and the neighboring population. DCRH is located in the Dire Dawa administration in eastern Ethiopia, located 515 km away from Addis Ababa and it annually serves around 193,485 people from nearby populations (1).

### Study population

Women who gave birth at the selected public hospitals and were within the six-week postpartum period were eligible to participate in this study. However, we excluded those who were unable to provide information due to severe physical or mental health conditions.

### Sample size determination

Since there had been no previous similar study that had used double population mean formula, the sample size was calculated by an online sample size calculator (https://www.surveysystem.com/sscalc.htm). So, it was calculated using a double population mean formula with a (95%) confidence level, (2%) margin of error, (80%), power, (*β* = 4.69, 95% CI: 2.63, 6.76) confidence interval from a previous study (22), and an average of 1,190 women who gave birth each month in the selected public hospitals as a source population. Then 10% of the calculated sample size was added to account for potential non-responses. And the final calculated sample size was 420.

### Sampling technique and procedure

We had proportionally allocated the calculated sample size (420) to each of the three selected hospitals based on their estimated number of monthly deliveries. The study participants were selected by a systematic random sampling technique. The sampling interval (k value) was calculated by dividing the total number of estimated monthly delivery reports by the required sample size, which is 1,190/420 ≈ 3. Finally, every third woman was recruited using her respective delivery registration number, and the first participant was selected by the lottery method.

### Data collection tool

The data collection tool was adapted by reviewing different literature (19, 22–24). This tool comprised sociodemographic characteristics, obstetrical factors, facility-related factors, and PCMC experiences of the participants. The data on PCMC items were collected using a validated person-centered maternity care scale adopted from previous studies (19, 22–24). The study participants' obstetrical data were extracted from the participants' medical records. The questionnaire was prepared first in English language and translated into local languages (Afan Oromo, Amharic, and Af-Somali) and back into English language by different language experts to ensure consistency.

### Data collection procedure

Six BSc Midwives, who do not work in the study area, collected the data under the supervision of the principal investigator and four MSc Midwives. They conducted interviewer-administered face-to-face exit interviews in the postnatal unit, using each participant's respective medical record.

### Data quality control

The questionnaire was pretested on 5% of the total sample size outside the study area to identify any ambiguity, check for consistency and acceptability, as well as to make necessary corrections before the actual data collection period. The questionnaire had a Cronbach's alpha of 0.89, indicating good internal reliability. Data collectors and supervisors were trained for one day by the principal investigator regarding the objectives of the study, data collection procedures, and the maintenance of confidentiality. The data were checked daily for completeness and consistency and, corrective measures were taken in a timely manner.

### Study variables

Person-centered maternity care (PCMC), which is composed of three domains, namely dignity and respect, communication and autonomy, and supportive care, was the outcome variable. Furthermore, sociodemographic characteristics (age, religion, marital status, residence, educational status, employment status, and average monthly family income), obstetrical factors (parity, ANC, number of institutional deliveries, mode of delivery, time of delivery, childbirth complications, and newborn outcome), and facility-related factors (sex of the main birth attendant and length of hospital stay) were the independent variables of this study.

### Measurements of outcome

PCMC is measured by the PCMC scale. The PCMC scale is a validated scale that comprises three domains (i.e., dignity and respect, communication and autonomy, and supportive care) (23, 24). There are a total of 30 items with each item having four-response options: i.e., 0- no, never, 1- yes, a few times, 2- yes, most of the time, and 3- yes, all the time. Negative items such as verbal abuse, physical abuse, and crowdedness were reversely coded to reflect a scale of 0 as the lowest level and 3 as the highest level. The total PCMC score is a summative score from the responses to the individual items, which ranges from 0 to 90 (19, 24). To enable easy comparison across the PCMC domains, the scores were rescaled and standardized to range from 0 to 100. Furthermore, verbal abuse is when a woman feels that the health providers shouted, scolded, insulted, threatened, or have spoken to her rudely. Physical abuse is when a woman feels that she is being treated roughly, such as being pushed, beaten, slapped, pinched, or physically restrained (24).

### Data management and analysis

Our data was entered into EpiData version 4.6 software and then exported to SPSS version 26 for further cleaning, coding, and analysis. Descriptive statistics was carried out to compute frequencies, proportions, means, and standard deviations. The normality assumption was assessed using a P-P plot and histogram. Linearity was checked using a scatter plot, and multicollinearity was checked using the Variance Inflation Factor (VIF). After creating dummy variables, simple and multiple linear regression analyses was used to determine the factors associated with PCMC. Those variables with *p*-value ≤0.25 in the simple linear regression were qualified to the final model. Unstandardized β coefficient, along with a 95% CI was used to report the strength of association and statistical significance was declared at a *P*-value of <0.05.

## Results

### Socio-demographic characteristics

Out of a total of 420 expected respondents, 412 provided complete responses, resulting in a response rate of 98.1%. More than half 242 (58.7%) of the study participants were from urban residences. The participants were in the age range of 16–42 years with a mean age of 25.83(±5.7) years. Nearly half 195 (47.3%) of these study participants had attended formal education (Table 1).

**Table 1:** Socio-demographic characteristics of the women who gave birth at selected public hospitals in eastern Ethiopia, 2022 (*n* = 412).

Variables	Category	Frequency (*n*)	Percentage
Residence	Urban	242	58.7
	Rural	170	41.3
Religion	Muslim	320	77.7
	Orthodox	65	15.8
	Protestant	27	6.6
Marital status	Married	410	99.5
	Divorced	1	0.2
	Widowed	1	0.2
Occupation	Housewife	246	59.7
	Self-employed	128	31.1
	Government employee	38	9.2
Educational status	No formal education	217	52.7
	Elementary	66	16
	Secondary	59	14.3
	Diploma	30	7.3
	Degree and above	40	9.7
Age in years	15–24	44	10.7
	25–34	248	60.2
	35–49	120	29.1
Average monthly family income	≤3,560 ETB	235	57.0
	>3,560 ETB	177	43.0

TB, Ethiopian Birr.

### The obstetrics characteristics of the study participants

Above three-fourths 316 (76.7%) of the participants were multiparous. The average parity of the participants was three. More than a quarter 123 (29.9%) of them had a history of abortion. There were 297 (72.1%) vaginal deliveries among the 412 participants. Of these, 65.3% were spontaneous vaginal deliveries, and 6.8% were assisted by operative vaginal delivery methods. Almost six-in-ten 254 (61.7%) of the overall participants had faced childbirth complications (Table 2).

**Table 2:** Obstetric characteristics of the women who gave birth at selected public hospitals in eastern Ethiopia, 2022 (*n* = 412).

Variables	Category	Frequency (*n*)	Percentage
Parity	Primiparous	96	23.3
	Multiparous	316	76.7
Abortion history	Yes	123	29.9
	No	289	70.1
ANC follow-up	Yes	297	72.1
	No	115	27.9
Number of ANC visits	Less than four	113	27.4
	Four or more	299	72.6
Place of ANC follow-up	Hospital	64	21.5
	Health center	193	65.0
	Private clinic	40	13.5
Number of institutional deliveries	≤ Two	258	62.6
	> Two	154	37.4
Mode of delivery	SVD	269	65.3
	Operative Vaginal delivery	28	6.8
	C/S	115	27.9
Type of C/S delivery	Elective	29	25.22
	Emergency	86	74.78
Sex of main delivery attendant	Male	181	43.9
	Female	137	33.3
	Both	94	22.8
Time of delivery	Day time	223	54.1
	Night time	189	45.9
Length of hospital stay	≤ Two days	208	50.5
	> Two days	204	49.5
Complication	Yes, for mother	109	26.5
	Yes, for neonate	62	15.0
	Yes, for both	83	20.1
	No, for both	158	38.3
Newborn outcome	Alive	354	85.9
	Dead	58	14.1

ANC, antenatal care; C/S, cesarean section; SVD, spontaneous vaginal delivery.

### Person-centered maternity care scale and subscales

**Figure 1:**
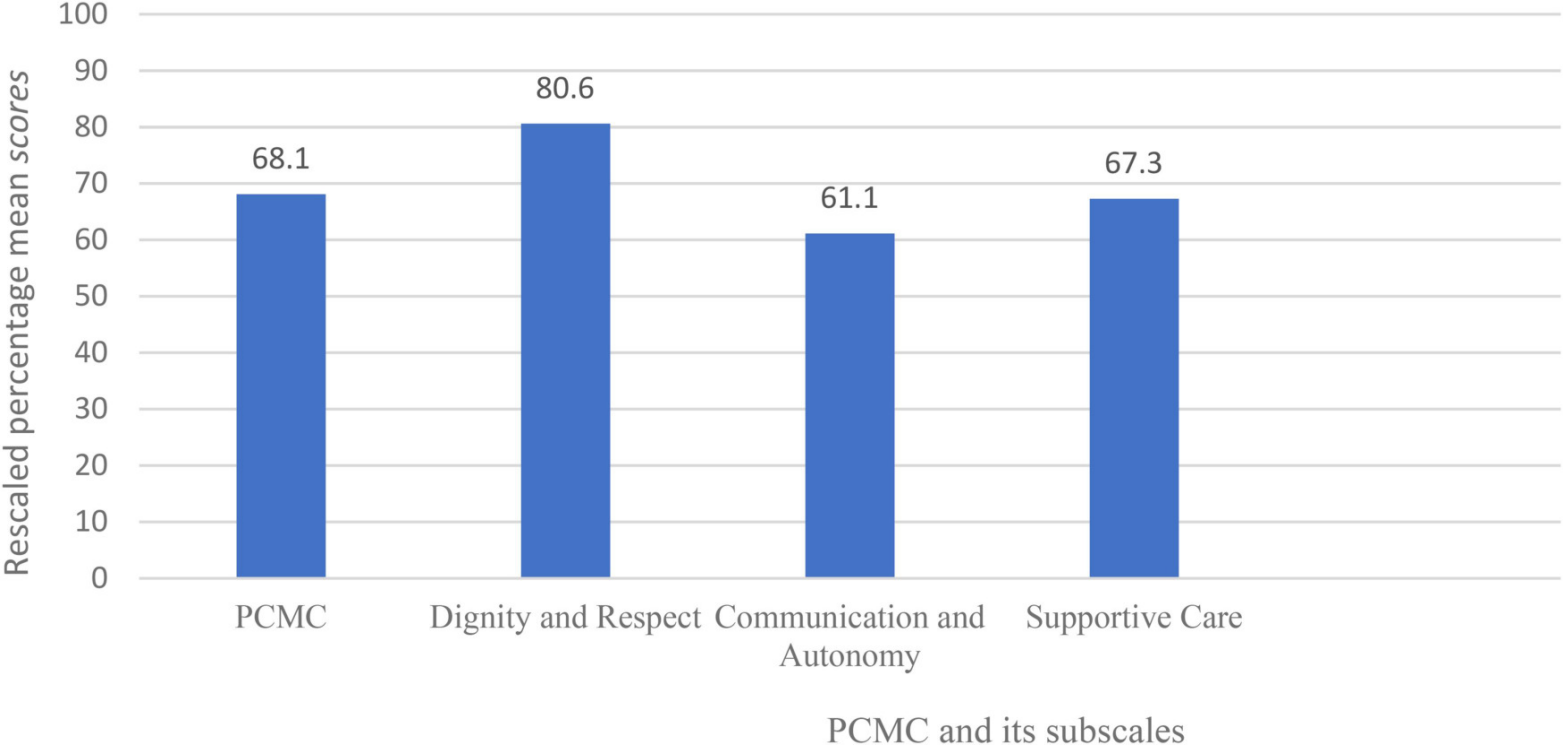
Rescaled percentage mean scores of person-centered maternity care and its subscales of women who gave birth at selected public hospitals in eastern Ethiopia, 2022 (*n* = 412).

The participants mean percentage score of PCMC was 68.1 (CI: 59.94, 62.66) with a standard deviation of ±14.1. From the subscales, the rescaled mean score of dignity and respect was 80.6%, communication and autonomy 61.1%, and 67.3% for supportive care (Figure 1).

### Dignity and respect

The percentage mean score of the study participants was 80.6 (±2.4). About two-thirds (66.6%, *n* = 272) of the total study participants felt that they were treated with respect all the time, and 240 (58.3%) of them reported that they were treated in a friendly manner all the time during their stay in the hospital. On the other hand, 61(14.8%) and 25 (6.1%) of women reported that they experienced verbal and physical abuse at least once during their stay at the hospital respectively (Table 3).

**Table 3:** Factors associated with PCMC of the women who gave birth at selected public hospitals of eastern, Ethiopia 2022 (*n* = 412).

Variables	Bivariable analysis	Multivariable analysis	
	β Coefficient (CI)	β Coefficient (CI)	*P*-value
Residence			
Urban	0	0	0
Rural	−5.92 (−8.65, −3.19)	−1.21 (−3.84, 1.42)	0.37
ANC follow-up			
No	0	0	0
Yes	7.85 (4.89, 10.81)	5.66 (2.79, 8.53)	<.001
Place of ANC			
Health center	0	0	0
Hospital	4.92 (1.17, 8.68)	1.94 (−1.59, 5.48)	0.28
Private clinic	6.0 (1.40, 10.59)	2.24(−2.09, 6.56)	0.31
Delivery time			
Day time	0	0	0
Night time	−3.21 (−5.95, −0.48)	−1.61 (−3.99, 0.79)	0.19
Faced pregnancy complication			
No	0	0	0
Yes	−11.58 (−14.17, −8.99	−7.01 (−9.88, −4.13)	<.001
Newborn outcome			
Dead	0	0	0
Alive	12.63 (8.88, 16.37)	7.59 (3.97, 11.20)	<.001
Length of hospital stay			
≤ Two days	0	0	0
> Two days	−9.34(−11.93, −6.75)	−4.08 (−6.79, −1.38)	0.003
Constant		61.82 (55.73, 67.92)	<.001

0, reference; CI, confidence interval; β, beta coefficient.

### Communication and autonomy

The percentage mean score of the participants was 61.1 (±6.2). More than three-fourths of the study participants 321 (77.9%) reported that providers never introduced themselves when they came to see them for the first time. More than half of the study participants 244 (59.2%) reported that providers called them by their names throughout their stay in the hospital. Slightly more than a quarter 108 (26.2%) women reported that providers had never explained the purpose of the examinations or procedures to them. Furthermore, one-third 134 (32.5%) of the participants reported that they were never asked for permission or consent during examinations (Appendix Table A1).

### Supportive care

The percentage mean score of the supportive care subscale of the participants was 67.3 (±7.3). Nearly half of the participants, 201 (48.8%) reported that they were not allowed to be with someone they wanted during labor and the majority of them 369 (89.6%) were without a companion during delivery. In addition, 219 (53.2%) of the women reported that providers supported their anxiety and fears all the time during the childbirth process and 254 (61%) of them felt providers took the best care for them all the time (Table 3).

### The factors that are associated with person-centered maternity care

From the overall variables entered into the simple linear regression analysis, residence, ANC follow-up, time of delivery, childbirth complications, newborn outcome, and length of hospital stay were eligible for the multiple linear regression analysis based on a *P*-value of less than 0.25. Ultimately, women who had ANC follow-up, faced childbirth complications, gave birth to a live newborn, and more than two days length of hospital stay remained significantly associated with person-centered maternity care (PCMC).

Women who'd had ANC follow-up had increased PCMC score by about 6 units compared women who had no ANC follow-up (*β* = 5.66, 95% CI: 2.79, 8.53). Women who faced childbirth complications had a lower PCMC score than those who didn`t face childbirth complication by 7 units (*β* = −7.01, 95% CI: −9.88, −4.13). Additionally, a live newborn outcome increased PCMC by about 8 units as compared to dead fetus newborn outcome (*β* = 7.59, 95% CI: 3.97, 11.20). By keeping all other variables constant, PCMC score was decreased by 4 units on those who had more than two days of hospital stay (*β* = −4.08, 95% CI: −6.79, −1.38) (Table 3).

## Discussion

This study assessed the status of PCMC and its associated factors during childbirth at three selected public hospitals in eastern Ethiopia. The percentage mean score of the PCMC scale was 68.1% (CI: 66.75, 69.45). This finding is consistent with a study conducted in Kenya, 66.9% (25). Nevertheless, this finding is slightly higher than studies conducted in Ethiopia (64.5%) (19), 51.6% in Ghana (25), 62.0% in India (25), and 47.1% in Sri Lanka (9). This discrepancy may be attributed to variations in the availability of hospital resources and obstetric and sociodemographic profiles of the participants including ANC follow-up, childbirth complications, and educational status. For instance, the study conducted in India reported that 79% of the participants faced pregnancy complications, which was found to be a significant factor for the decrement of PCMC, as identified by this study and other previous studies (9, 20, 26, 27).

Women who'd had ANC follow-up had higher PCMC than those who had no ANC follow-up, which is consistent with a study conducted in Addis Ababa (28). The reason could be that, those who do have ANC follow-up are more likely to establish positive establish interactions with the healthcare providers and become familiar with the hospital environment. Furthermore, health providers counsel them on birth plan preparedness and any possible complications they may face during the childbirth process to help them prepare. And this results in a pleasant and cooperative intervention. In addition, ANC follow-up plays an essential role in health promotion, detection, and appropriate management of any pregnancy risk factors, which is a vital factor for a positive childbirth experience and PCMC.

Women who faced childbirth complications had lower PCMC score than those who did not face childbirth complications, which is consistent with the studies conducted in Addis Ababa, Bahir Dar, and Harar (20, 26, 28). The reason might be because women experiencing complications often require more medical interventions, which can shift the focus of care from a person-centered approach to a more clinical or procedure-oriented approach. This shift can lead to less attention being paid to the individual needs and preferences of the woman. Additionally, healthcare providers may be under increased time constraints when dealing with complications, which can limit their ability to provide emotional support (27, 29, 30). Furthermore, childbirth complications often induce functional limitations and anxiety, affecting the emotional well-being of the mother and potentially resulting in unpleasant client-provider interaction and a negative birth experience (31, 32).

Giving birth to a live newborn increased PCMC as compared to its counterpart. This finding is in line with the findings of studies conducted in Dessie and Kenya (10, 19). This could be because a positive birth experience is often associated with positive emotions such as joy, relief, and satisfaction. This positive emotional state can enhance the woman's perception of her care experience, making her more likely to report higher levels of engagement and person-centered maternity care. Positive outcomes can also foster an environment where providers are more attentive to the woman's preferences and needs. When outcomes are positive, healthcare providers may be more inclined to focus on the holistic needs of the woman, including emotional support and personalized care (33).

Conversely, women who had more than two days of hospital stay reported lower PCMC scores compared to their counterparts. This finding is supported by studies conducted in Dessie town, Ethiopia, and Kenya (19, 24). A possible explanation is that prolonged hospitalization may increase discomfort due to facility conditions, compromise privacy, and contribute to emotional stress. Moreover, extended stays are often related to maternal and/or neonatal complications, and all of these factors can decrease PCMC level.

### The strengths and limitations of this study

As the first study conducted in this area, it uncovered the status of PCMC and its associated factors. However, a limitation of this study is that it was conducted only in public hospitals, which means it did not address the status of PCMC in private facilities within the study area. Moreover, the data related to PCMC were collected solely from participants' self-reports, making them prone to social desirability and recall bias. To help participants recall information, the time frame was limited to the six weeks postpartum period. Also, participants were informed and assured that their responses will remain anonymous and confidential, to encourage more honest responses. Furthermore, data collectors were trained to avoid leading questions in order to reduce the pressure to respond in socially desirable ways.

## Conclusion

The study revealed that the mean person-centered maternity care score of the participants was higher than that in previous studies. Regarding the subscales, dignity and respect had the highest score, while the communication and autonomy subscale had the lowest score. Women who had ANC follow-up, faced childbirth complications, gave birth to a live newborn, and had a hospital stay of more than two days were significantly associated with PCMC. Therefore, we strongly suggest that strengthening of timely initiation and adherence to antenatal care follow-up, as well as early detection and appropriate management of childbirth complications, would greatly improve PCMC.

## Data Availability

The raw data supporting the conclusions of this article will be made available by the authors, without undue reservation.

## Appendix

**Table A1:** PCMC sub-scale items of the women who gave birth at selected public hospitals in eastern Ethiopia, 2022 (*n* = 412).

Items	No, never (%)	Yes, few times (%)	Yes, most of the time (%)	Yes, all the time (%)
Dignity and Respect items				
Providers treated me with respect	4 (1)	26 (6.3)	110 (26.7	272 (66.0)
Providers treated in friendly a manner	27 (6.6)	46 (11.2)	99 (24.0)	240 (58.3)
I experienced Verbal abuse	321 (77.9)	61 (14.8)	19 (4.6)	11 (2.7)
I experienced physical abuse	365 (88.6)	25 (6.1)	16 (3.9)	6 (1.5)
Audio privacy was ensured	126 (30.6)	52 (12.6)	88 (21.4)	146 (35.4)
I feel my health care information was or will kept confidential	27 (6.6)	24 (5.8)	100 (24.3)	261 (63.3)
Communication and autonomy items				
Providers introduced themselves to me	321 (77.9)	53 (12.9)	28 (6.8)	10 (24)
Providers called me by my name	5 (1.2)	24 (5.8)	139 (33.7)	244 (59.2)
Providers spoke to me in a language I can understand	6 (1.5)	24 (5.8)	113 (27.4)	269 (65.3)
The procedures were explained to me	108 (26.2)	46 (11.2)	85 (20.6)	173 (42.0)
The purpose of medications was explained to me	95 (23.1)	40 (9.7)	70 (17.0)	207 (50.2)
I was able to ask questions	33 (8.0)	61 (14.8)	89 (21.6)	229 (55.6)
I was involved in my healthcare decision	53 (12.9)	62 (15.0)	75 (18.2)	222 (53.9)
Providers asked me for consent before procedures	134 (32.5)	91 (22.1)	69 (16.7)	118 (28.6)
I was able to be on my preferred delivery position	123 (29.9)	64 (15.5)	74 (18.0)	151 (36.7)
Supportive care items				
I was allowed to have companion during labor	201 (48.8)	56 (13.6)	90 (21.8)	65 (15.8)
I was allowed to have companion during delivery	369 (89.6)	10 (2.4)	13 (3.2)	20 (4.9)
Providers talked to me about my feeling	26 (6.3)	53 (12.9)	102 (24.8)	231 (56.1)
Providers supported my anxiety and fears	47 (11.4)	58 (14.1)	88 (21.4)	219 (53.2)
Providers managed my pain	79 (19.2)	62 (15.0)	58 (14.1)	213 (51.7)
Providers paid attention when I need them	36 (8.7)	51 (12.4)	79 (19.2)	246 (59.7)
Providers take the best for me	23 (5.6)	43 (10.4)	92 (22.3)	254 (61.7)
I completely trust the health providers	33 (8.0)	50 (12.1)	97 (23.5)	232 (56.3)
There were enough health providers	43 (10.4)	41 (10.0)	93 (22.6)	235 (57.0)
The hospital was crowded	102 (24.8)	100 (24.3)	90 (21.3)	120 (29.3)
There was water in the hospital	79 (19.2)	63 (15.3)	118 (28.6)	152 (36.9)
There was electricity in the hospital	5 (1.2)	4 (1.0)	76 (18.4)	327 (79.4)
I feel safe in the hospital	2 (0.5)	8 (1.9)	73 (17.7)	329 (79.9)
Waiting time before being seen by the health care provider	Very long (%)	Somewhat long (%)	Short (%)	Very short (%)
	19 (4.6)	72 (17.5)	88 (21.4)	233 (56.6)
General sanitation of the hospital	Very dirty (%)	Dirty (%)	Clean (%)	Very clean (%)
	11 (2.7)	78 (18.9)	151 (36.7)	33 (8.0)
